# Correction to the Entropy of a Charged Rotating Accelerated Black Hole Due to Lorentz Invariance Violation

**DOI:** 10.3390/e28010062

**Published:** 2026-01-05

**Authors:** Cong Wang, Hui-Ying Wang, Shu-Zheng Yang

**Affiliations:** 1 College of Physics and Electronic Engineering, Qilu Normal University, Jinan 250300, China; 2 College of Physics and Astronomy, China West Normal University, Nanchong 637002, China; szyangcwnu@126.com

**Keywords:** charged rotating accelerated black hole, LIV correction, Bekenstein–Hawking entropy

## Abstract

In the spacetime of a charged rotating accelerated black hole, the dynamics equations of fermions and bosons are modified by Lorentz invariance violation (LIV). The correction effects of LIV on the quantum tunneling radiation of this black hole are investigated. New expressions for the quantum tunneling rate, Hawking temperature, and Bekenstein–Hawking entropy of this black hole, which depend on the charge parameter and acceleration parameter, are derived, incorporating LIV correction terms. The physical implications of these results are discussed in depth.

## 1. Introduction

In the four fundamental forces of physics, the strong interaction, weak interaction, and electromagnetic interaction are described by the Standard Model of particle physics, while the gravitational interaction is described by general relativity. Gravitational interaction is much weaker than the other three forces. Moreover, gravity is a non-renormalizable theory, and thus, there remains a fundamental contradiction between gravity and quantum theory that has yet to be resolved. In order to study quantum gravity theory and the Grand Unified Theory in physics, a series of meaningful studies have been conducted on modified gravity theories, including string theory, Einstein-aether gravity theory, and Horava–Lifshitz gravity theory. Some quantum gravity theories suggest that Lorentz symmetry may be broken at high energies, which implies that by considering Lorentz invariance violation (LIV), one can study the renormalization problem of gravity. In high-energy physics, Lorentz symmetry breaking indicates that Lorentz symmetry is restored at low energies and satisfies the results of current scientific experiments. This theory includes the Horava–Lifshitz gravity theory and the Einstein-aether gravity theory. These gravity theories are modifications of Einstein’s general relativity. The study of the effects of Lorentz symmetry breaking can be divided into three aspects: one is the investigation of gravitational experiments at short distances. Models of the differences between gravity and the other three forces predict deviations from Newton’s law of gravitation at short distances [1]. Existing short-distance gravitational experiments have placed constraints on the Lorentz invariance violation coefficients at the level of $10^{-8}\;m^{2}$. Research in this area is still ongoing. In flat spacetime, Lorentz symmetry is global, whereas in curved spacetime, Lorentz symmetry is local. Studies of quantum gravity theories suggest that Lorentz invariance is violated at the Planck scale, which opens a window for exploring new physics while detecting Lorentz violation. Experimental studies on detecting Lorentz-breaking in curved spacetime can facilitate research on dark energy and astronomical observations related to it. Another aspect is modifying the Einstein gravitational action by adding a Lorentz-breaking correction term. By studying the solutions of the gravitational field equations, new expressions for the spacetime metric containing the Lorentz-breaking correction coefficients can be obtained. This type of modification is a correction to the curved spacetime background [2]. The third method for studying the LIV effects in curved spacetime is to modify the action of scalar and spinor fields in curved spacetime and then variationally calculate the corresponding particle dynamics equations for the action with the Lorentz-breaking correction term. Based on this, the quantum tunneling radiation characteristics of black holes and related topics are investigated [3,4]. For spinor fields, another method of modifying the spin-1/2 fermion equation is to directly add an LIV correction term to the left-hand side of the original Dirac equation. Based on this, the modified spin-1/2 fermion dynamics equations and related topics can be studied according to different characteristics of curved spacetime. The research presented in this paper adopts this modification method to investigate the correction to the entropy of a charged rotating accelerated black hole. This is a topic that has not been deeply explored yet. Both the accelerated motion of black holes and the accelerated expansion of the universe are areas worthy of further study. The mechanism of the universe’s accelerated expansion is explained by the presence of dark energy, and thus, research has been conducted on spacetime metric features and related topics in the case where a quintessence field exists around black holes [5,6]. Recent astronomical observations suggest the presence of an axion dark energy model in the universe. These new findings indicate that the universe undergoes both accelerated expansion and contraction, which implies that the accelerated expansion and evolution of the universe remain areas worthy of further study. The mechanisms of accelerated motion in black holes and related research are another important topic of interest. The acceleration of black holes is related to cosmic strings. In recent years, there has been research on the thermodynamics of accelerated black holes and related topics [7,8,9,10]. Reference [3] also studied the thermodynamic evolution characteristics of charged non-rotating accelerated black holes. The novel aspect of this paper is the consideration of the LIV and Einstein-aether theories in the correction of the entropy of a charged rotating accelerated black hole. As a result, a series of new and meaningful findings will be obtained.

In the following Section 2, we will present the correction results for the entropy of a charged rotating accelerated black hole due to LIV. In Section 3, we will introduce the correction to the scalar particle dynamics equation in the spacetime of a charged rotating accelerated black hole according to the Einstein-aether theory, as well as the resulting correction to the black hole’s entropy. Section 4 of the paper will provide an in-depth discussion of the results obtained in this study.

## 2. LIV Corrections to the Temperature and Entropy of a Charged Rotating Accelerated Black Hole

A charged rotating accelerated black hole is described by the C-metric as [10,11,12]

(1) $$\begin{array}{c} ds^{2}=\frac{1}{H^{2}}\left\{\frac{f(r)}{\sum }{\left[\frac{dt}{\alpha }-a\sin\theta \frac{d\phi }{K}\right]}^{2}-\frac{\sum }{f(r)}dr^{2}-\frac{\sum r^{2}}{g(\theta )}d\theta^{2}-\frac{g\left(\theta \right)\sin^{2}\theta }{\sum r^{2}}{\left[\frac{adt}{\alpha }-\left(r^{2}+a^{2}\right)\frac{d\phi }{K}\right]}^{2}\right\} \end{array}$$

In this C-metric, *M* represents the mass scale of the black hole, *A* denotes the acceleration, and *K* represents the conical deficit of the spacetime. The parameter *ℓ* corresponds to the negative cosmological constant, given by $\sigma =-\frac{3}{\ell^{2}}$, and α represents the parameter responsible for the rescaling of *t*. Equation (1) contains

(2) $$\begin{array}{cc} f(r) & =\;\left(1-A^{2}r^{2}\right)\left(1-\frac{2m}{r}+\frac{a^{2}+q^{2}}{r^{2}}\right)+\frac{r^{2}+a^{2}}{\ell^{2}}\\ g(\theta ) & =\;1+2Am\cos\theta +\left(\Xi -1\right)\cos^{2}\theta \\ \Sigma & =\;1+\frac{a^{2}}{r^{2}}\cos^{2}\theta \\ H & =\;1+Ar\cos\theta \\ \Xi & =\;1+\ell^{2}A^{2}-\frac{a^{2}}{\ell^{2}}\left(1-A^{2}\ell^{2}\right) \end{array}$$

The non-zero components of the electromagnetic potential $A_{\mu }$ generated by the charge *q* of this black hole at the event horizon $r_{+}$ are respectively:

(3) $$\begin{array}{cc} A_{t} & =\;\frac{qr_{+}}{\left(r_{+}^{2}+a^{2}\right)\alpha }\\ A_{\phi } & =\;\frac{qr_{+}a\sin^{2}\theta }{\left(r_{+}^{2}+a^{2}\right)\alpha } \end{array}$$

Based on Equations (1) and (2), the corresponding determinant of the metric $g_{\mu \nu }$ can be calculated as:

(4) $$\begin{array}{c} g=-\frac{{\left(r^{2}+a^{2}\cos^{2}\theta \right)}^{2}}{\alpha^{2}K^{2}H^{8}}\sin^{2}\theta \end{array}$$

From Equations (1), (2) and (4), the contravariant metric tensor can be calculated as:

(5) $$\begin{array}{c} g^{u\nu }=\left(\begin{array}{cccc} g^{tt} & 0 & 0 & g^{t\phi }\\ 0 & g^{rr} & 0 & 0\\ 0 & 0 & g^{\theta \vartheta } & 0\\ g^{\phi t} & 0 & 0 & g^{\phi \phi } \end{array}\right) \end{array}$$

where,

(6) $$\begin{array}{cc} g^{tt} & =\;\sum H^{2}\alpha^{2}\frac{{\left(r^{2}+a^{2}\right)}^{2}-a^{2}\sin^{2}\theta r^{2}f\left(r\right)}{f\left(r\right)g\left(\theta \right){\left(r^{2}+a^{2}\cos^{2}\theta \right)}^{2}}\\ g^{rr} & =\;-\frac{f\left(r\right)H^{2}}{\sum }\\ g^{\theta \theta } & =\;-\frac{g\left(\theta \right)H^{2}}{\sum r^{2}}\\ g^{t\phi } & =\;g^{\phi t}=\sum H^{2}a\alpha K\frac{r^{2}f\left(r\right)-g\left(\theta \right)\left(r^{2}+a^{2}\right)}{f\left(r\right)g\left(\theta \right){\left(r^{2}+a^{2}\cos^{2}\theta \right)}^{2}}\\ g^{\phi \phi } & =\;-\frac{\sum H^{2}K^{2}\left[r^{2}f\left(r\right)-a^{2}\sin^{2}\theta g\left(\theta \right)\right]}{\sin^{2}\theta {\left(r^{2}+a^{2}\cos^{2}\theta \right)}^{2}f\left(r\right)g\left(\theta \right)} \end{array}$$

Equation (6) described non-zero components of $g^{\mu \nu }$. The event horizon of this black hole is determined by the following null hypersurface equation.

(7) $$\begin{array}{c} g^{\mu \nu }\frac{\partial f}{\partial x^{\mu }}\frac{\partial f}{\partial x^{\nu }}=0 \end{array}$$

Substituting Equation (6) into Equation (7) gives the equation that the event horizon $r_{+}$ of this black hole satisfies:

(8) $$\begin{array}{c} f\left(r_{+}\right)=\left(1-A^{2}r_{+}^{2}\right)\left(1-\frac{2m}{r_{+}}+\frac{a^{2}+q^{2}}{r_{+}^{2}}\right)+\frac{r_{+}^{2}+a^{2}}{\ell^{2}}=0 \end{array}$$

From this equation, it is clear that the event horizon $r_{+}$ of this black hole is related to M,A,a,q,ℓ. The curved spacetime described by Equation (1) possesses a Killing vector $\partial_{\phi }$. Since g(θ) is related to cosθ, the two-dimensional line element $ds_{\theta \phi }^{2}$ at the north pole ($\theta_{+}=0$) and the south pole ($\theta_{-}=\pi$) on the event horizon are not the same. This difference is what makes this accelerated black hole unique. To explain the acceleration mechanism of this black hole, it is necessary to clarify the cause of its accelerated motion. Physically speaking, the energy-momentum tensor of physical entities that can interact with the event horizon of this black hole satisfies the condition $T_{0}^{0}∼T_{r}^{r}$ in the local region. The physical entity that satisfies this condition is a cosmic string. The energy-momentum of the cosmic string is dominated by its mass per unit length, and the string’s tension is numerically equal to this energy [13]. The gravitational effect of a cosmic string does not generate long-range curvature in spacetime but induces a global conical defect on the spatial section perpendicular to the string. Therefore, we can consider the cosmic string as essentially a localized conical deficit in spacetime. It is this conical deficit that drives the accelerated motion of this black hole. This is the fundamental reason for the accelerated motion of the black hole, with the acceleration parameter denoted by *A*. From Equation (2), it can be seen that *A* affects f(r), and this effect manifests as an influence on the cosmological constant. The effect of *A* on g(θ) primarily reflects the range of values for Aℓ. Therefore, we can impose the condition Aℓ<1, which better reflects the variation in g(θ). According to Equations (1) and (2), the regularity of the metric at a pole requires that $K_{\pm }$ be as follows:

(9) $$\begin{array}{c} K_{\pm }=g\left(\theta_{\pm }\right)=1\pm 2Am+\ell^{2}A^{2}\left(1+\frac{a^{2}}{\ell^{2}}\right)-\frac{a^{2}}{\ell^{2}} \end{array}$$

If θ=0 is fixed with $K_{\pm }=K$ along the two-pole axis, given by [7,9,14]

(10) $$\begin{array}{c} \delta =2\pi \left[1-\frac{g(\theta )}{K_{+}}\right]=\frac{8\pi mA}{1+2Am+\ell^{2}A^{2}\left(1+\frac{a^{2}}{\ell^{2}}\right)-\frac{a^{2}}{\ell^{2}}} \end{array}$$

The deficit angle of the conical defect is interpreted as being due to the cosmic string. We can consider the tensions of the strings along each axis as $\mu_{\pm }=\delta_{\pm }/8\pi$, that is

(11) $$\begin{array}{c} \mu_{\pm }=\frac{1}{4}\left[1-\frac{\Xi \pm 2mA}{K}\right] \end{array}$$

Here, the + sign corresponds to the north pole, and the − sign corresponds to the south pole. $\mu_{\pm }$ is interpreted as a cosmic string emerging from the black hole, causing it to accelerate. Knowing the reason for the accelerated motion of this black hole and the basic characteristics of the curved spacetime, we can study the thermodynamic evolution of this charged, rotating, accelerated black hole.

In the spacetime of this charged, rotating, accelerated black hole, the equation for spin-1/2 fermions is the Dirac equation, which is given by:

(12) $$\begin{array}{c} \left(\gamma^{\mu }D_{\mu }+\frac{m_{D}}{\hbar }\right)\psi =0 \end{array}$$

where,

(13) $$\begin{array}{c} D_{\mu }=\partial_{\mu }+\frac{i}{\hbar }eA_{\mu }+\Omega_{\mu } \end{array}$$

where $\Omega_{\mu }$ is the spin connection in the curved spacetime. It is given by $\Omega_{\mu }=\frac{i}{2}\Gamma_{\mu }^{\alpha \beta }\Pi_{\alpha \beta },\;\Pi_{\alpha \beta }=\frac{i}{4}\left[\gamma^{\alpha },\gamma^{\beta }\right]$. In the spacetime of the charged, rotating, accelerated black hole described by Equations (1) and (2), and considering LIV corrections, we propose modifying Equation (12) as [15]:

(14) $$\begin{array}{c} \left(\gamma^{\mu }D_{\mu }+\frac{m_{D}}{\hbar }-\zeta \hbar \gamma^{t}D_{t}\gamma^{j}D_{j}\right)\psi =0 \end{array}$$

The term $\zeta \hbar \gamma^{t}D_{t}\gamma^{j}D_{j}\psi$ in Equation (14) represents the LIV correction written according to the contraction rules for differential and tensor free indices in general relativity. The coupling constant ζ≪1. In fact, Keuglov considered LIV corrections in flat spacetime and introduced a correction term $\zeta \hbar \gamma^{t}D_{t}\gamma^{j}D_{j}\psi$, which modified the Dirac equation [15]. When extending this to curved spacetime, the LIV correction term we choose must be as shown in Equation (14). $m_{D}$ in Equation (14) is the mass of the Dirac particle. ψ is the wave function of the spin-1/2 fermions. $\gamma^{\mu }$ is the gamma matrix in the curved spacetime described by Equation (1). The $\gamma^{\mu }$ matrices are required to satisfy the following commutation relation:

(15) $$\begin{array}{c} \gamma^{\mu }\gamma^{\nu }+\gamma^{\nu }\gamma^{\mu }=2g^{\mu \nu }I \end{array}$$

The $\gamma^{\mu }$ matrices that satisfy Equations (1) and (15) are given as follows:

(16) $$\begin{array}{cc} \gamma^{t} & =\;\sqrt{g^{tt}}\left(\begin{array}{cc} I & 0\\ 0 & -I \end{array}\right)\\ \gamma^{r} & =\;\sqrt{g^{rr}}\left(\begin{array}{cc} 0 & \sigma^{1}\\ \sigma^{1} & 0 \end{array}\right)\\ \gamma^{\theta } & =\;\sqrt{g^{\theta \theta }}\left(\begin{array}{cc} 0 & \sigma^{2}\\ \sigma^{2} & 0 \end{array}\right)\\ \gamma^{\phi } & =\;\frac{g^{t\phi }}{\sqrt{g^{tt}}}\left(\begin{array}{cc} I & 0\\ 0 & -I \end{array}\right)+\sqrt{\frac{g^{tt}g^{\phi \phi }-{\left(g^{t\phi }\right)}^{2}}{g^{tt}}}\left(\begin{array}{cc} 0 & \sigma^{3}\\ \sigma^{3} & 0 \end{array}\right) \end{array}$$

The Pauli matrices $a^{i}$ are expressed as follows:

(17) $$\begin{array}{c} \sigma^{1}=\left(\begin{array}{cc} 0 & 1\\ 1 & 0 \end{array}\right),\;\sigma^{2}=\left(\begin{array}{cc} 0 & -i\\ i & 0 \end{array}\right),\;\sigma^{3}=\left(\begin{array}{cc} 1 & 0\\ 0 & -1 \end{array}\right) \end{array}$$

According to the WKB semiclassical approximation theory, we can express the wave function ψ in Equation (14) in terms of the particle action *S* as follows:

(18) ψ=ABeiSℏ

Substituting Equation (18) into Equation (14), we can obtain:

(19) iγμ∂μS+eAμ+mD+ζgtϕ∂tS+eAt∂ϕS+eAϕAB=0

In Equation (19), $\gamma^{\mu }$ is a 4 × 4 matrix, and *A* and *B* must be 2-dimensional spinors. In the above equation, $\gamma^{\mu }$ is a Hermitian matrix. The solution of the matrix equation can only be guaranteed to have physical significance if the matrix is Hermitian. In Equation (19), there is a term that contains the imaginary unit *i*, and another term related to $g^{t\phi }$. In order to solve for the particle action *S*, we need to correctly choose a transformation related to $g^{t\phi }$ and $\gamma^{\mu }$ based on the characteristics of the stationary spacetime. Therefore, we need to introduce a matrix transformation based on the characteristics of the stationary, axisymmetric black hole spacetime as follows:

(20) $$\begin{array}{c} \begin{array}{c} \Gamma^{\mu }=\gamma^{\mu }-i\zeta \left[\left(\partial_{t}S+eA_{t}\right)\gamma^{t}\gamma^{\mu }+\left(\partial_{\phi }S+eA_{\phi }\right)\gamma^{\phi }\gamma^{\mu }\right] \end{array} \end{array}$$

From this, it can be concluded that:

(21) $$\begin{array}{c} \Gamma^{\mu }\Gamma^{\nu }=\gamma^{\mu }\gamma^{\nu }-i2\zeta \left[\left(\partial_{t}S+eA_{t}\right)\gamma^{t}\gamma^{\mu }\gamma^{\nu }+\left(\partial_{\phi }S+eA_{\phi }\right)\gamma^{\phi }\gamma^{\mu }\gamma^{\nu }\right]+O\left(\zeta^{2}\right) \end{array}$$

(22) $$\begin{array}{c} \Gamma^{\mu }\left(\partial_{\mu }S+eA_{\mu }\right)=\gamma^{\mu }\left(\partial_{\mu }S+eA_{\mu }\right)-i\zeta \left[\left(\partial_{t}S+eA_{t}\right)\gamma^{t}+\left(\partial_{\phi }S+eA_{\phi }\right)\gamma^{\phi }\right]\gamma^{\mu }\left(\partial_{\mu }S+eA_{\mu }\right) \end{array}$$

Substituting Equation (22) into Equation (19), we obtain the spin-1/2 fermion equation in the spacetime of the charged, rotating, accelerated black hole as:

(23) iΓμ∂μS+eAμ−ζgtt∂tS+eAt2−ζgϕϕ∂ϕS+eAϕ2−ζgtϕ∂tS+eAτ∂ϕS+eAϕ+mDAB=0

This is a matrix equation, and in fact, it is an eigenmatrix equation. The $\Gamma^{\mu }$ in Equation (23) is related to $\gamma^{\mu }$. From this equation, we see that the AB is a 4 × 1 matrix and both A and B are 2-component spinors. The wave function ψ represented by the semiclassical WKB approximation theory is meaningful. The four terms inside the left-hand bracket of Equation (23) that are independent of $\Gamma^{\mu }$ are all related to scalars. In the matrix equation Equation (23), $m_{D}$ can actually be expressed as $m_{D}\left(\begin{array}{cc} I & 0\\ 0 & I \end{array}\right)$, where $I=\left(\begin{array}{cc} 1 & 0\\ 0 & 1 \end{array}\right)$ is a 2 × 2 identity matrix, and the other three terms can be expressed in a similar way. The first term inside the left-hand bracket of the equation no longer has free indices because the upper index μ and the lower index μ have already been contracted. Therefore, this term can also be expressed as a term involving a 4 × 4 identity matrix. We denote the term inside the left-hand bracket of Equation (23) as *G*, we have

(24) $$\begin{array}{c} G\left(\begin{array}{cc} I & 0\\ 0 & I \end{array}\right)\left(\begin{array}{c} A\\ B \end{array}\right)=0 \end{array}$$

That is

(25) $$\begin{array}{c} \left(\begin{array}{cc} GI & 0\\ 0 & GI \end{array}\right)\left(\begin{array}{c} A\\ B \end{array}\right)=0 \end{array}$$

This matrix equation is an eigenmatrix equation. For this equation to have a non-trivial solution, we must require that the determinant of the matrix $\left(\begin{array}{cc} GI & 0\\ 0 & GI \end{array}\right)$ is zero, that is,

(26) $$\begin{array}{c} \det\left(\begin{array}{cc} GI & 0\\ 0 & GI \end{array}\right)=0 \end{array}$$

From this equation, it can be concluded that

(27) $$\begin{array}{cc} G^{4} & =\;\left\{i\Gamma^{\mu }\left(\partial_{\mu }S+eA_{\mu }\right)-\zeta \left[g^{tt}{\left(\partial_{t}S+eA_{t}\right)}^{2}+g^{\phi \phi }{\left(\partial_{\phi }S+eA_{\phi }\right)}^{2}\right.\right.\\ \; & {\left.\left.-g^{t\phi }\left(\partial_{t}S+eA_{t}\right)\left(\partial_{\phi }S+eA_{\phi }\right)\right]+m_{D}\right\}}^{4}=0 \end{array}$$

So, we obtain the following equation:

(28) $$\begin{array}{c} i\Gamma^{\mu }\left(\partial_{\mu }S+eA_{\mu }\right)-\zeta \left[g^{tt}{\left(\partial_{t}S+eA_{t}\right)}^{2}+g^{\phi \phi }{\left(\partial_{\phi }S+eA_{\phi }\right)}^{2}-g^{t\phi }\left(\partial_{t}S+eA_{t}\right)\left(\partial_{\phi }S+eA_{\phi }\right)\right]+m_{D}=0 \end{array}$$

The first term on the left-hand side of Equation (28) contains the imaginary unit *i*. To solve this equation, we use the relationship between $\Gamma^{\mu }$ and $\gamma^{\mu }$, as shown in Equation (20). By multiplying both sides of Equation (28) by $i\Gamma^{\nu }$ and utilizing the relation between $\gamma^{\mu }$ and $g^{\mu \nu }$ given in Equation (15), we obtain:

(29) $$\begin{array}{c} g^{\mu \nu }\left(\partial_{\mu }S+eA_{\mu }\right)\left(\partial_{\nu }S+eA_{\nu }\right)-2\zeta^{\prime }\left[g^{tt}{\left(\partial_{t}S+eA_{t}\right)}^{2}+g^{\phi \phi }{\left(\partial_{\phi }S+eA_{\phi }\right)}^{2}+g^{t\phi }\left(\partial_{t}S+eA_{t}\right)\left(\partial_{\phi }S+eA_{\phi }\right)\right]+m_{D}^{2}=0 \end{array}$$

From Equation (28) to Equation (29), $O(\zeta^{2})$ is neglected, and we set $\zeta =\frac{\zeta^{\prime }}{m_{D}}$, $2\zeta m_{D}=2\zeta^{\prime }$, where $\zeta^{\prime }\ll 1$ is the correction parameter. It should be noted that in Equations (19), (23), (28) and (29), the free indices μ and ν take values 0, 1, 2, 3. When the upper index μ (or ν) is contracted with the lower index μ (or ν), it results in a scalar term (with no free indices). Therefore, each term on the left-hand side of Equations (19), (23), (28) and (29) is a scalar. Equation (29) is the semiclassical modified form of the dynamical equation for spin-1/2 fermions in the spacetime of a charged, rotating, accelerated black hole, expressed in terms of the particle action, including LIV corrections. The equation without the LIV correction term is the Hamilton-Jacobi equation. Therefore, Equation (29) is an equation that can be used to study the characteristics of stationary black hole quantum tunneling radiation. Substituting Equation (6) into Equation (29) gives:

(30) $$\begin{array}{cc} \; & \left(1-2\zeta^{\prime }\right){\left(\partial_{t}S+eA_{t}\right)}^{2}\Sigma H^{2}\alpha^{2}\frac{{\left(r^{2}+a^{2}\right)}^{2}-a^{2}\sin^{2}\theta r^{2}f\left(r\right)}{f(r)}\\ \; & -\left(1-2\zeta^{\prime }\right){\left(\partial_{\phi }S+eA_{\phi }\right)}^{2}\frac{\Sigma H^{2}K^{2}\left[r^{2}f\left(r\right)-a^{2}\sin^{2}\theta g\left(\theta \right)\right]}{\sin^{2}\theta f\left(r\right)}\\ \; & +\left(1-2\zeta^{\prime }\right)\left(\partial_{t}S+eA_{t}\right)\left(\partial_{\phi }S+eA_{\phi }\right)\frac{\Sigma H^{2}\alpha aK\left[r^{2}f\left(r\right)-g\left(\theta \right)\left(r^{2}+a^{2}\right)\right]}{f(r)}\\ \; & -{\left(r^{2}+a^{2}\cos^{2}\theta \right)}^{2}g\left(\theta \right)\Sigma \left[\frac{f\left(r\right)H^{2}}{\Sigma }{\left(\frac{\partial S}{\partial r}\right)}^{2}-\frac{H^{2}g\left(\theta \right)}{\Sigma r^{2}}{\left(\frac{\partial S}{\partial \theta }\right)}^{2}+m_{D}^{2}\right]=0 \end{array}$$

In the curved spacetime described by Equations (1) and (2), with the basic characteristics of a stationary spacetime and a Killing vector $\partial_{\phi }$, the particle action *S* in Equation (30) can be separated into the following form:

(31) $$\begin{array}{c} S=-\omega t+R(r)+\Theta (\theta )+j\phi \end{array}$$

Substituting Equation (31) into Equation (30) and performing variable separation, let the constant introduced during the separation process be denoted as $Y_{0}$. We can then obtain the equation satisfied by the radial action R(r) for the spin-1/2 fermion in the curved spacetime described by Equation (1) as:

(32) $$\begin{array}{cc} {\left[r^{2}f\left(r\right)\frac{dR}{dr}\right]}^{2} & =\;\left(1-2\zeta^{\prime }\right)\left\{{\left(\omega -eA_{t}\right)}^{2}\alpha^{2}{\left(r^{2}+a^{2}\right)}^{2}+a^{2}K^{2}j^{2}+2\left(\omega -eA_{t}\right)j\alpha aK\left[r^{2}f\left(r\right)-\left(r^{2}+a^{2}\right)\right]\right\}\\ \; & +\;f\left(r\right)\left(r^{4}m_{D}^{2}+Y_{0}\right) \end{array}$$

As $r\to r_{+}$, we have $f(r_{+})=0$. Therefore, from Equation (32), we obtain:

(33) $$\begin{array}{c} {\left.\frac{dR^{\pm }}{dr}\right|}_{r\to r_{+}}=\pm \frac{\alpha \left(r_{+}^{2}+a^{2}\right){\left(1-2\zeta^{\prime }\right)}^{\frac{1}{2}}\left(\omega -\omega_{0}\right)}{{\left.r_{+}^{2}f\left(r\right)\right|}_{r\to r_{-}}} \end{array}$$

where,

(34) $$\begin{array}{c} \omega_{0}=\frac{eqr_{+}+aKj}{\alpha \left(r_{+}^{2}+a^{2}\right)} \end{array}$$

By applying the residue theorem and integrating both sides of Equation (33), we obtain the radial action $R^{\pm }$ as:

(35) $$\begin{array}{c} R^{\pm }=\pm i\pi \frac{\alpha \left(r_{+}^{2}+a^{2}\right){\left(1-2\zeta^{\prime }\right)}^{\frac{1}{2}}\left(\omega -\omega_{0}\right)}{f^{\prime }\left(r_{+}\right)r_{+}^{2}} \end{array}$$

where,

(36) $$\begin{array}{c} f^{\prime }\left(r_{+}\right)=\frac{2m}{r_{+}^{2}}-\frac{2\left(a^{2}+q^{2}\right)}{r_{+}^{3}}+\frac{2r_{+}}{\ell^{2}}-2A^{2}r_{+}^{2}\left(r_{+}-m\right) \end{array}$$

According to the theory of black hole quantum tunneling radiation, the quantum tunneling rate for spin-1/2 fermions at the event horizon $r_{+}$ of this charged, rotating, accelerated black hole is given by [16,17]:

(37) Γ∼exp−2ImS±=exp−2ImR±=exp−4παr+2+a2f′r+r+2(1−2ζ′)12ω−ω0=exp−ω−ω0TH

where $T_{H}$ is the Hawking temperature of the event horizon of this black hole, given by:

(38) $$\begin{array}{c} T_{H}=\frac{f^{\prime }\left(r_{+}\right)r_{+}^{2}}{4\pi \alpha \left(r_{+}^{2}+a^{2}\right){\left(1-2\zeta^{\prime }\right)}^{\frac{1}{2}}}\approx \frac{f^{\prime }\left(r\right)r_{+}^{2}}{4\pi \alpha \left(r_{+}^{2}+a^{2}\right)}\left(1+\zeta^{\prime }\right) \end{array}$$

where the higher-order small quantity $O(\zeta^{\prime 2})$ is neglected. Obviously, LIV has an impact on $T_{H}$. $T_{H}$ is independent of θ. According to the zeroth law of black hole thermodynamics, the surface gravity κ of a stationary black hole is a constant, and $T_{H}=\frac{\kappa }{2\pi }$, where $\kappa =\frac{f^{\prime }\left(r_{+}\right)r_{+}^{2}}{\alpha (r_{+}^{2}+a^{2})}$ is constant. According to the first law of black hole thermodynamics, there is an inherent connection between the black hole temperature and entropy. The first law of thermodynamics for accelerated black holes with conical deficits has been studied in [7,8,18,19,20]. Reference [21] investigates the modified Bekenstein–Hawking entropy for a charged, non-rotating accelerated black hole. In contrast to these studies, this paper focuses on the modified Bekenstein–Hawking entropy for charged, rotating, accelerated black holes and its associated physical significance. We denote the tensions of the strings along each axis as $u_{\pm }$ and the thermodynamic length associated with the string tensions as $\lambda_{\pm }$, and use $S_{BH}$ to represent the Bekenstein–Hawking entropy of this black hole. Include the conical deficit as a change and introduce the conjugate chemical potential, use tools from holographic renormalization to properly calculate the various charges of the slowly accelerating black hole spacetime the mathematical expression for the first law of thermodynamics related to this black hole is as follows [8,18]:

(39) $$\begin{array}{c} dM=T_{H}dS_{BH}^{\prime }+\Phi dQ+\Omega dJ+\lambda_{+}d\mu_{+}+\lambda_{-}d\mu_{-}+VdP \end{array}$$

In Equation (39), the mass of the black hole is determined as $M=\frac{m}{K\Xi }{\left[(\Xi +a^{2}\ell^{2}\Xi )\right]}^{\frac{1}{2}}$. The accelerating black hole also obeys a Smarr relation M=2(TS+ΩJ−PV)+ΦQ. The value of α, satisfying the first law and the Smarr relation, is given by $\alpha =\frac{{\left[\left(\Xi +a^{2}/\ell^{2}\right)\left(1-A^{2}\ell^{2}\Xi \right)\right]}^{\frac{1}{2}}}{1+a^{2}A^{2}}$ [8,10,18]. Where *V* is the thermodynamic volume of this black hole. $J=\frac{ma}{K^{2}}$, $\Phi =\Phi_{t}=\frac{qr_{+}}{(r_{+}^{2}+a^{2})\alpha }$. From Equation (39), it follows that

(40) $$\begin{array}{c} S_{BH}^{\prime }=\int \frac{dM-\Phi dQ-\Omega dJ-\lambda_{+}d\mu_{+}-\lambda_{-}d\mu_{-}-VdP}{T_{H}}={\left(1-2\zeta^{\prime }\right)}^{\frac{1}{2}}S_{BH}\approx S_{BH}\left(1+\zeta^{\prime }\right) \end{array}$$

where $S_{BH}^{\prime }$ is the Bekenstein–Hawking entropy of this black hole without LIV corrections. To calculate $S_{BH}^{\prime }$, we first consider the 2-dimensional line element obtained from Equation (1) as follows:

(41) $$\begin{array}{c} dS_{\theta \phi }=-\frac{r_{+}^{2}+a^{2}\cos^{2}\theta }{g(\theta )}d\theta^{2}-\frac{g\left(\theta \right)\sin^{2}\theta {\left(r_{+}^{2}+a^{2}\right)}^{2}}{r_{+}^{2}+a^{2}\cos^{2}\theta }\frac{d\phi^{2}}{K^{2}} \end{array}$$

From Equation (41), the area of the event horizon of this black hole is given by:

(42) $$\begin{array}{c} A_{s}=4\pi \frac{r_{+}^{2}+a^{2}}{K^{2}} \end{array}$$

Therefore, $S_{BH}=\frac{\pi }{K}\left(r_{+}^{2}+a^{2}\right)$. The entropy $S_{BH}^{\prime }$ in Equation (40) can be expressed as:

(43) $$\begin{array}{c} S_{BH}^{\prime }={\left(1-2\zeta^{\prime }\right)}^{\frac{1}{2}}\frac{\pi \left(r_{+}^{2}+a^{2}\right)}{K}\approx \frac{\pi (r_{+}^{2}+a^{2})}{K}\left(1-\zeta^{\prime }\right) \end{array}$$

Here, the higher-order small quantity $O(\zeta^{\prime 2})$ is neglected. Where *K* is as shown in Equation (9). From Equation (43), it can be seen that LIV introduces a correction to the entropy of this black hole.

In addition to the LIV corrections, we can also consider the effects of quantum corrections. For this, let $\tilde{\omega_{0}}=\omega -\omega_{0}$, and use *ℏ* perturbation theory to express the energy and radial action of the spin-1/2 fermion as follows:

(44) $$\begin{array}{c} \tilde{\omega }=\tilde{\omega_{0}}+\sum_{i=1}\hbar^{i}\tilde{\omega_{i}} \end{array}$$

(45) $$\begin{array}{c} {\tilde{R}}^{\pm }=R_{0}^{\pm }+\sum_{j=1}\hbar^{i}R_{j}^{\pm } \end{array}$$

Here, $R_{0}^{\pm }$ represents the radial action in semiclassical theory, as shown in Equation (35). $\tilde{\omega_{0}}$ corresponds to $\left(\omega -\omega_{0}\right)$ in Equations (33), (35) and (37). From Equation (45), we have $\tilde{R_{1}^{\pm }}=R_{0}^{\pm }+\hbar^{1}R_{1}^{\pm },\tilde{R_{2}^{\pm }}=R_{0}^{\pm }+\hbar^{1}R_{1}^{\pm }+\hbar^{2}R_{2}^{\pm },\cdots$. Using Equations (33), (44) and (45), we can obtain the equation associated with $R_{0}^{\pm }$ as follows:

(46) $$\begin{array}{c} {\left.\frac{dR_{0}^{\pm }}{dr}\right|}_{r\to r_{+}}=\pm \frac{\alpha \left(r_{+}^{2}+a^{2}\right){\left(1-2\zeta^{\prime }\right)}^{\frac{1}{2}}}{{\left.r_{+}^{2}f\left(r\right)\right|}_{r\to r_{+}}}\tilde{\omega_{0}} \end{array}$$

(47) $$\begin{array}{c} {\left.dR_{1}^{\pm }\right|}_{r\to r_{+}}=\pm \frac{\alpha \left(r_{+}^{2}+a^{2}\right){\left(1-2\zeta^{\prime }\right)}^{\frac{1}{2}}}{{\left.r_{+}^{2}f\left(r\right)\right|}_{r\to r_{+}}}\tilde{\omega_{1}} \end{array}$$

(48) $$\begin{array}{c} {\left.\frac{dR_{2}^{\pm }}{dr}\right|}_{r\to r_{+}}=\pm \frac{\alpha \left(r_{+}^{2}+a^{2}\right){\left(1-2\zeta^{\prime }\right)}^{\frac{1}{2}}}{{\left.r_{+}^{2}f\left(r\right)\right|}_{r\to r_{+}}}\tilde{\omega_{2}} \end{array}$$

Similarly, one can write down the equation satisfied by $\tilde{R_{l}^{\pm }}$. Obviously, there exists a definite relationship between $R_{i}^{\pm }$ and $R_{i-1}^{\pm }$. Let $R_{i}^{\pm }/R_{i-1}^{\pm }=\alpha_{i}^{\prime }=\alpha_{i}/S_{BH}$, from Equations (35) and (45)–(48), we obtain:

(49) $$\begin{array}{c} \tilde{R^{\pm }}=R_{0}^{\pm }+\sum_{i=1}\hbar^{i}\alpha_{i}^{\prime }R_{0}^{\pm }=\pm i\pi \frac{\alpha \left(r_{+}^{2}+a^{2}\right){\left(1-2\zeta^{\prime }\right)}^{\frac{1}{2}}}{f^{f}\left(r_{+}\right)r_{+}^{2}}\left(1+\sum_{i=1}\hbar^{i}\alpha_{i}/S_{BH}\right)\left(\omega -\omega_{0}\right) \end{array}$$

From this, we can derive the quantum tunneling rate for the spin-1/2 fermion at the event horizon of this black hole as:

(50) Γ˜∼exp−2ImR±˜=exp−ω−ω0T0˜

where,

(51) $$\begin{array}{c} \tilde{T_{H}}=\frac{f^{\prime }\left(r_{+}\right)r_{+}^{2}}{4\pi \alpha \left(r_{+}^{2}+a^{2}\right){\left(1-2\zeta^{\prime }\right)}^{\frac{1}{2}}}{\left(1+\sum_{i=1}\hbar^{i}\alpha_{i}^{\prime }/S_{BH}\right)}^{-1}\left(\omega -\omega_{0}\right) \end{array}$$

After the $\hbar^{i}$ corrections, the Bekenstein–Hawking entropy can be expressed as:

(52) $$\begin{array}{c} \tilde{S_{BH}}=\int \frac{dM-\Phi dQ-\Omega dJ-\lambda_{+}d\mu_{+}-\lambda_{-}d\mu_{-}-VdP}{{\tilde{T}}_{H}}=S_{BH}+\hbar^{1}\alpha_{1}\ln S_{BH}+\ldots \end{array}$$

where $\alpha_{1}=\alpha_{1}^{\prime }S_{BH},\alpha_{1}^{\prime }=R_{1}^{\pm }/R_{0}^{\pm }$. In $S_{BH}$, the LIV correction term is represented by the coefficient $\zeta^{\prime }$, while in $\tilde{S_{BH}}$, the correction terms include both the LIV correction coefficient and the $\hbar^{i}$ correction terms. Equation (52) represents the new expression for the modified Bekenstein–Hawking entropy of the charged, rotating, accelerated black hole, as described by Equations (1) and (2).

It should be further clarified that, according to the literature [15], the Dirac equation for spin-1/2 particles in flat spacetime with LIV corrections is given by $\left(\gamma_{\mu }\partial_{\mu }+m-i\left(\gamma_{\varphi }\partial_{t}\gamma_{i}\partial_{i}\right)\right)\psi \left(x\right)$, where $\partial_{\mu }=\frac{\partial }{\partial x_{\mu }}=\left(\frac{\partial }{\partial x_{i}},\frac{\partial }{i\partial t}\right)$, and $x_{0}=t$. When considering $\psi \left(x\right)=\psi \left(p\right)e^{i(px-p_{0}x_{0})}$, we obtain $p_{0}^{2}=p^{2}+m^{2}-L^{2}p_{0}^{2}p^{2}$. When L=0, the well-known Lorentz dispersion relation is recovered. Both general relativity and quantum field theory are based on the Lorentz dispersion relation. The study of LIV has prompted research into modified forms of the fermion dynamics equations in both flat and curved spacetime, as well as related topics. The above research method is not suitable for studying the dynamics equations of bosons. In the next section, we will investigate the modifications to the boson dynamics equations in curved spacetime as described by Equations (1)–(3), along with the related topics.

## 3. Lorentz-Breaking and the Scalar Field Equation and Black Hole Entropy in the Spacetime of a Charged Rotating Accelerating Black Hole

The previous section discussed the LIV corrections to the dynamical equations of spinor field particles, and used the spin-1/2 fermion as an example to study the modified entropy of a charged, rotating, accelerating black hole. Since the effects of LIV can be studied in curved spacetime, we can more generally express Lorentz violation as Lorentz-breaking. This allows us to introduce Lorentz-breaking correction terms into the particle action in different gravitational fields, and, based on this, apply the variational principle to obtain the modified particle dynamics equations. The following section will use a scalar particle with zero spin as an example to study the modification of the entropy of the charged, rotating, accelerating black hole. LIV indicates that Lorentz symmetry is broken under high-energy conditions. The Einstein-aether theory is a gravitational theory that incorporates Lorentz-breaking. By introducing an aether-like vector field $u^{\mu }$, the action of Einstein’s gravitational field is modified. Then, using the variational principle, the modified form of the scalar field equation is derived. Based on this, the modified dynamical equation for scalar particles (bosons) with spin zero is obtained using the WKB approximation theory, as follows [21].

(53) $$\begin{array}{c} \left(g^{\mu \nu }+\sigma u^{\mu }u^{\nu }\right)\left(\frac{\partial S}{\partial x^{\mu }}+eA_{\mu }\right)\left(\frac{\partial S}{\partial x^{\nu }}+eA_{\nu }\right)+m^{2}=0 \end{array}$$

This is a semiclassical dynamical equation for spin-0 Bosons. The correction term $\sigma u^{\mu }u^{\nu }\left(\frac{\partial }{\partial x^{\mu }}+eA_{\mu }\right)\left(\frac{\partial S}{\partial x^{2}}+eA_{r}\right)$ corresponds to the Lorentz-breaking correction term in the Einstein-aether gravity theory. For Bosons with other spins, a separate study is required to investigate the modified form of their dynamical equations. In Equation (14), σ is a coupling constant and σ≪1. The reference [21] uses this equation to study the quantum tunneling radiation characteristics of a class of black holes, but the quantum tunneling radiation features in curved spacetime described by Equations (1)–(3) have not been studied yet. According to Equations (1) and (2), the chosen $u^{\mu }$ must satisfy the condition $u^{\mu }u_{\mu }=\mathrm{const}$. The four components of $u_{\mu }$ are selected as follows:

(54) $$\begin{array}{c} u_{t}=\frac{c_{t}}{\sqrt{g^{tt}}},u_{t}u^{t}=u_{t}u_{t}g^{tt}=c_{t}^{2}\\ u_{r}=\frac{c_{r}}{\sqrt{g^{rr}}},u_{r}u^{r}=u_{r}u_{r}g^{rr}=c_{r}^{2}\\ u_{\theta }=\frac{c_{\theta }}{\sqrt{g^{\theta \theta }}},u_{\theta }u^{\theta }=u_{\theta }u_{\theta }g^{\theta \theta }=c_{\theta }^{2}\\ u_{\phi }=\frac{c_{\phi }}{\sqrt{g^{\phi \phi }}},u_{\phi }u^{\phi }=u_{\phi }u_{\phi }g^{\phi \phi }=c_{\phi }^{2} \end{array}$$

From this equation, we can see that $u^{\mu }u_{\mu }=2c_{t}^{2}+c_{r}^{2}+c_{\theta }^{2}+2c_{\phi }^{2}=C$ (constant). Therefore, the chosen $u^{\mu }$ is correct. By substituting Equations (6) and (54) into Equation (53), the dynamical equation for the spin-zero boson in the spacetime of the charged, rotating, accelerating black hole is given by:

(55) $$\begin{array}{c} \left(1+\sigma c_{t}^{2}\right)\Sigma H^{2}\alpha^{2}\frac{{\left(r^{2}+a^{2}\right)}^{2}-a^{2}\sin^{2}\theta r^{2}f\left(r\right)}{f\left(r\right)g\left(\theta \right){\left(r^{2}+a^{2}\cos^{2}\theta \right)}^{2}}{\left(\frac{\partial S}{\partial t}+eA_{t}\right)}^{2}-\left(1+\sigma c_{r}^{2}\right)\frac{f\left(r\right)H^{2}}{\Sigma }{\left(\frac{\partial S}{\partial r}\right)}^{2}\\ -\;\left(1+\sigma c_{\theta }^{2}\right)\frac{g\left(\theta \right)H^{2}}{\Sigma r^{2}}{\left(\frac{\partial S}{\partial \theta }\right)}^{2}-\left(1+\sigma c_{\phi }^{2}\right)\frac{\Sigma H^{2}K^{2}\left[r^{2}f\left(r\right)-a^{2}\sin^{2}\theta g\left(\theta \right)\right]}{\sin^{2}\theta {\left(r^{2}+a^{2}\cos^{2}\theta \right)}^{2}f\left(r\right)g\left(\theta \right)}{\left(\frac{\partial S}{\partial \phi }+eA_{\phi }\right)}^{2}\\ +\;2\left(1+\sigma c_{t}^{2}\right)\Sigma H^{2}a\alpha K\frac{r^{2}f\left(r\right)-g\left(\theta \right)\left(r^{2}+a^{2}\right)}{f\left(r\right)g\left(\theta \right){\left(r^{2}+a^{2}\cos^{2}\theta \right)}^{2}}\left(\frac{\partial S}{\partial t}+eA_{t}\right)\left(\frac{\partial S}{\partial \phi }+eA_{\phi }\right)+m^{2}\\ +\;2\sigma \left[u^{t}u^{r}\left(\frac{\partial S}{\partial r}\right)\left(\frac{\partial S}{\partial t}+eA_{t}\right)+u^{t}u^{\theta }\left(\frac{\partial S}{\partial \theta }\right)\left(\frac{\partial S}{\partial t}+eA_{t}\right)+u^{r}u^{\phi }\left(\frac{\partial S}{\partial r}\right)\left(\frac{\partial S}{\partial \phi }+eA_{\phi }\right)\right.\\ \left.+\;u^{r}u^{\theta }\left(\frac{\partial S}{\partial r}\right)\left(\frac{\partial S}{\partial \theta }\right)+u^{\theta }u^{\phi }\left(\frac{\partial S}{\partial \theta }\right)\left(\frac{\partial S}{\partial \phi }+eA_{\phi }\right)\right]=0 \end{array}$$

Separate the variables in this equation. Isolate the equation involving (t,r), let $\tilde{Y_{o}}$ is the constant introduced during the separation process. Consider that both $\frac{1}{\sqrt{g^{tt}}}$ and $\frac{1}{\sqrt{g^{\phi \phi }}}$ are related to $\sqrt{f(r)}$, and that ${\left.f(r)\right|}_{r\to r_{+}}=0$. Therefore, we examine the case where $r\to r_{+}$. Substituting Equation (31) into Equation (55), we obtain

(56) $$\begin{array}{cc} {\left.\left(1+\sigma c_{r}^{2}\right){\left[r^{2}f\left(r\right)\frac{dR}{dr}\right]}^{2}\right|}_{r\to r_{+}} & =\;\left(1+\sigma c_{t}^{2}\right)\left[{\left(\omega -{\left.eA_{t}\right|}_{r\to r_{+}}\right)}^{2}\alpha^{2}\left(r_{+}^{2}+a^{2}\right)+a^{2}K^{2}j^{2}\right.\\ \; & \left.-\;2\left(\omega -{\left.eA_{t}\right|}_{r\to r_{+}}\right)\alpha aKj\left(r_{+}^{2}+a^{2}\right)\right]+{\left.f(r)\right|}_{r\to r_{+}}\left(r_{+}^{4}m^{2}+\tilde{Y_{o}}\right) \end{array}$$

From this equation, we obtain:

(57) $$\begin{array}{c} {\left.\frac{dR^{\pm }}{dr}\right|}_{r\to r_{+}}=\pm \frac{\alpha \left(r_{+}^{2}+a^{2}\right)\left(\omega -\omega_{0}\right)}{{\left.r_{+}^{2}f\left(r\right)\right|}_{r\to r_{+}}}{\left(\frac{1+\sigma c_{t}^{2}}{1+\sigma c_{r}^{2}}\right)}^{\frac{1}{2}} \end{array}$$

where $\omega_{0}$ is consistent with Equation (33). Applying the residue theorem to solve Equation (57), we obtain:

(58) $$\begin{array}{c} R^{\pm }=\pm i\pi \frac{\alpha \left(r_{+}^{2}+a^{2}\right)}{f\left(r_{+}\right)r_{+}^{2}}{\left(\frac{1+\sigma c_{t}^{2}}{1+\sigma c_{r}^{2}}\right)}^{\frac{1}{2}}\left(\omega -\omega_{0}\right) \end{array}$$

where $f^{\prime }\left(r_{+}\right)$ is as shown in Equation (36). According to the quantum tunneling radiation theory for black holes, we obtain the quantum tunneling rate for the spin-zero boson at the event horizon of the charged, rotating, accelerating black hole as:

(59) Γ∼exp−2ImS±=exp−2ImR±=exp−4παr+2+a2f′r+r+21+σct21+σcr212ω−ω0=exp−ω−ω0TH˜

where,

(60) $$\begin{array}{c} \tilde{T_{H}^{\prime }}=\frac{f^{\prime }\left(r_{+}\right)r_{+}^{2}}{4\pi \alpha \left(r_{+}^{2}+a^{2}\right)}{\left(\frac{1+\sigma c_{r}^{2}}{1+\sigma c_{t}^{2}}\right)}^{\frac{1}{2}}\approx \frac{f^{\prime }\left(r_{+}\right)r_{+}^{2}}{4\pi \alpha \left(r_{+}^{2}+a^{2}\right)}\left[1+\frac{1}{2}\sigma \left(c_{r}^{2}-c_{t}^{2}\right)\right] \end{array}$$

Here, higher-order small quantities $O(\sigma^{2})$ are neglected. This is the result of introducing the aether-like vector field $u^{\mu }$ according to the Einstein-aether gravitational theory and the correction to the Hawking temperature and quantum tunneling rate of this black hole. Clearly, the components $u^{t}$ and $u^{r}$ of $u^{\mu }$ have an influence on the tunneling radiation of this black hole. Once the Lorentz-breaking effects are taken into account, the quantum tunneling radiation of this black hole will be significantly affected by the LIV corrections. Based on the corresponding expression of the first law of thermodynamics for this black hole (Equation (39)), we can obtain the corrected Bekenstein–Hawking entropy of this black hole under the Einstein-aether theory as

(61) $$\begin{array}{c} \tilde{S_{BH}^{\prime }}={\left(\frac{1+\sigma c_{t}^{2}}{1+\sigma c_{r}^{2}}\right)}^{\frac{1}{2}}S_{BH} \end{array}$$

According Equation (42) $S_{BH}=A_{s}/4$. The corrections to this black hole are related to $\sigma ,c_{t},c_{r}$. The above results regarding the tunneling radiation of spin-zero bosons are derived within the semiclassical theory. To further investigate the quantum corrections, the perturbative theory involving *ℏ* needs to be considered for further refinement. Using the same *ℏ*-expansion method as in the previous section, we can derive the corrected result for the entropy of this black hole, which is logarithmically related, i.e.,

(62) $$\begin{array}{c} \tilde{S_{BH}^{*}}=\tilde{S_{BH}^{\prime }}+\hbar^{\prime }\alpha_{1}\ln\tilde{S_{BH}^{\prime }}+\cdots \end{array}$$

The $S_{BH}$ in Equation (61) is related to the $S_{BH}$ in Equation (52), as shown in Equation (43).

## 4. Discussion

In previous related papers, only the LIV-corrected black hole entropy for charged accelerating black holes was studied. The difference in this paper is that it investigates the LIV-corrected entropy for charged, rotating, and accelerating black holes. The results presented in this paper are novel. In the above research, we have separately studied the quantum tunneling radiation characteristics of spin-1/2 fermions and spin-zero scalar particles at the event horizon of a charged, rotating, accelerating black hole spacetime. We derived a new expression for the corrected Bekenstein–Hawking entropy of this black hole. These specific expressions include both the results under the semiclassical theory and the corresponding quantum corrected results. The acceleration mechanism of the charged, rotating, accelerating black hole discussed in this paper holds special significance. Since the curved spacetime described by Equations (1) and (2) exhibits a stationary characteristic, the rotation and acceleration of this black hole are inherently stable. The key distinguishing feature of this black hole compared to other types of stationary black holes is that its acceleration is caused by cosmic strings, and this special acceleration mechanism warrants further investigation. It is certain that, based on the work in Reference [3], we have enriched the corrected results for the Hawking temperature and Bekenstein–Hawking entropy of the charged, rotating, accelerating black hole through the discussions in this paper. This contributes to a deeper understanding of the thermodynamic evolution of black holes and related topics. Furthermore, it should be noted that the methods outlined above can be applied to study the quantum tunneling radiation characteristics and the corrections of related physical quantities in different stationary curved spacetimes. We can also modify the action of the spinor field by adding correction terms related to Lorentz-breaking and revise the fermion dynamical equations. On this basis, we can study the quantum tunneling radiation and its corrected expressions for various types of stationary black holes. For nonstationary black holes, when studying LIV corrections, the methods described above cannot be directly applied to the corrections of physical quantities like black hole entropy. We must reconsider the specific form of the transformation matrix. This is the topic we need to further study. In future research, we will conduct meaningful studies on the impact of Lorentz-breaking on the spacetime background and related topics.

## Data Availability

Data is contained within the article.

## References

[1] Adelberger E.G., Gundlach J.H., Heckel B.R., Hoedl S., Schlamminger S. (2009). Torsion balance experiments: A low-energy frontier of particle physics. Prog. Part. Nucl. Phys..

[2] Carleo A., Lambiase G., Mastrototaro L. (2022). Energy extraction via magnetic reconnection in Lorentz breaking Kerr–Sen and Kiselev black holes. Eur. Phys. J. C.

[3] Wang C., Tan X., Zhang J., Li R., Yang S.Z. (2024). Correction of Kerr-Sen Black Hole Temperature and Entropy by Lorentz Invariance Violation. Phys. Scr..

[4] Liu Y.Z., Wang C., Zhang J., Yang S.Z. (2025). Correction of Bekenstein–Hawking entropy of Kiselev black holes surrounded by quintessence owing to Lorentz breaking. Eur. Phys. J. C.

[5] Saghafi S., Nozari K. (2022). Shadow behavior of the quantum-corrected Schwarzschild black hole immersed in holographic quintessence. J. Hologr. Appl. Phys..

[6] Kiselev V.V. (2002). Quintessence and black holes. arXiv.

[7] Appels M., Gregory R., Kubizňák D. (2016). Thermodynamics of accelerating black holes. Phys. Rev. Lett..

[8] Anabalón A., Gray F., Gregory R., Kubiznak D., Mann R.B. (2013). Thermodynamics of charged, rotating, and accelerating black holes. arXiv.

[9] Gregory R. (2017). Accelerating black holes. arXiv.

[10] Gregory R., Scoins A. (2019). Accelerating black hole chemistry. Phys. Lett. B.

[11] Kinnersley W., Walker M. (1970). Uniformly accelerating charged mass in general relativity. Phys. Rev. D.

[12] Plebanski J.F., Demianski M. (1976). Rotating, charged, and uniformly accelerating mass in general relativity. Ann. Phys..

[13] Vilenkin A. (1985). Cosmic strings and domain walls. Phys. Rep..

[14] Krtouš P. (2005). Accelerated black holes in an anti-de Sitter universe. Phys. Rev. D—Part Fields Gravit. Cosmol..

[15] Kruglov S.I. (2012). Modified Dirac equation with Lorentz invariance violation and its solutions for particles in an external magnetic field. Phys. Lett. B.

[16] Parikh M.K., Wilczek F. (2000). Hawking Radiation As Tunneling. Phys. Rev. Lett..

[17] Parikh M.K. (2002). New coordinates for de Sitter space and de Sitter radiation. Phys. Lett. B.

[18] Anabalón A., Appels M., Gregory R., Kubiznak D., Mann R.B., Övgün A. (2018). Holographic thermodynamics of accelerating black holes. arXiv.

[19] Astorino M. (2016). CFT Duals for Accelerating Black Holes. Phys. Lett. B.

[20] Astorino M. (2017). Thermodynamics of regular accelerating black holes. arXiv.

[21] Wang C., Zhang J., Yang S.-Z. (2025). Entropy Modifications of Charged Accelerating Anti-de Sitter Black Hole. Entropy.

